# Mental health problems and socioeconomic disadvantage: a controlled household study in rural Ethiopia

**DOI:** 10.1186/s12939-019-1020-4

**Published:** 2019-07-31

**Authors:** Yohannes Hailemichael, Charlotte Hanlon, Kebede Tirfessa, Sumaiyah Docrat, Atalay Alem, Girmay Medhin, Abebaw Fekadu, Crick Lund, Dan Chisholm, Damen Hailemariam

**Affiliations:** 1 Department of Reproductive Health and Health Services Management, School of Public Health, College of Health Sciences, Addis Ababa University, Addis Ababa, Ethiopia; 2 Department of Health Economics, Policy and Management, College of Health Sciences, Jimma University, Jimma, Ethiopia; 3 Department of Psychiatry, School of Medicine, College of Health Sciences, Addis Ababa University, Addis Ababa, Ethiopia; 4 Centre for Global Mental Health, Health Service and Population Research Department, Institute of Psychiatry, Psychology and Neuroscience, King’s College, London, UK; 5 Alan J. Flisher Centre for Public Mental Health, Department of Psychiatry and Mental Health, University of Cape Town, Cape Town, South Africa; 6 Aklilu-Lemma Institute of Pathobiology, Addis Ababa University, Addis Ababa, Ethiopia; 7 Department of Mental Health and Substance Abuse, World Health Organization, Geneva, Switzerland; 8 Department of Global Health & Infection, Brighton and Sussex Medical School, Brighton, UK; 9 Centre for Innovative Drug Development and Therapeutic Trials for Africa (CDT-Africa), College of Health Sciences, Ababa University, Addis Ababa, Ethiopia; 10 Centre for Affective Disorders, Department of Psychological Medicine, Institute of Psychiatry, Psychology and Neuroscience, King’s College, London, UK

**Keywords:** Socio-economic status, Severe mental disorder, Depression

## Abstract

**Background:**

There is a lack of high quality population-based studies from low- and middle-income countries examining the relative economic status of households with and without a member with a mental health problem. The aim of the study was to explore the socio-economic status of households with a person with severe mental disorder (SMD; psychosis or bipolar disorder) or depression compared to households without an affected person.

**Methods:**

A population-based, comparative, cross-sectional household survey was conducted in Sodo district, south Ethiopia, between January and November 2015. Two samples were recruited, each with its own comparison group. Sample (1): households of 290 community-ascertained persons with a clinician-confirmed diagnosis of SMD and a comparison group of 289 households without a person with SMD. Sample (2): households of 128 people who attended the primary health care centre and who were identified by primary care staff as having a probable diagnosis of depressive disorder; and comparison households of 129 patients who attended for other reasons and who did not receive a diagnosis of depression. Household socioeconomic status (household income, consumption and asset-based wealth) was assessed using a contextualized version of theWorld Health Organization (WHO) Study on global Ageing and adult health (SAGE) questionnaire. Each disorder group (SMD and depression) was further divided into higher and lower disability groups on the basis of median score on the WHO Disability Assessment Schedule.

**Results:**

Households of a person with SMD who had higher disability were more likely to have a poorer living standard (no toilet facility; *p* < 0.001). Having a reliable source of regular income was significantly lower in households of a person with SMD (*p* = 0.008) or depression (*p* = 0.046) with higher disability than the comparison group. Households of persons with SMD with higher disability earned less (*p* = 0.005) and owned significantly fewer assets (*p* < 0.001) than households without SMD. Households including persons with depression who had higher disability had lower income (*p* = 0.042) and reduced consumption (*p* = 0.048).

**Conclusions:**

Households with a member who had either SMD or depression were socioeconomically disadvantaged compared to the general population. Moreover, higher disability was associated with worse socio-economic disadvantage. Prospective studies are needed to determine the direction of association. This study indicates a need to consider households of people with SMD or depression as a vulnerable group requiring economic support alongside access to evidence-based mental healthcare.

**Electronic supplementary material:**

The online version of this article (10.1186/s12939-019-1020-4) contains supplementary material, which is available to authorized users.

## Introduction

In the seminal paper of Faris and Dunham in 1939 [1], an inverse relationship between socioeconomic status and rates of schizophrenia was identified. It has been argued that mental health problems make an important contribution to inequality in socioeconomic status across populations [2–4]. People living with severe mental disorders (SMD), including psychotic disorders, such as schizophrenia and bipolar disorder, are thought to drift into poverty as a consequence of factors such as reduced income, increased medical and transport costs, and lost productivity [5]. The economic effects of SMD extend beyond the individual with a mental health problem to adversely impact household income [6]. Household members may devote time to provide care and support, diminishing their own opportunities to work [7], in turn affecting their income, and thus further increasing the risk of household poverty [6]. Depression, on the other hand, is perceived to be less associated with enduring disability compared to SMD, although has been associated with increased health care expenditure and reduced work productivity [8–10]. However, in several population-based studies conducted in high-income countries, there was weak and inconsistent evidence of an association between common mental disorders (including depression) and low socio-economic status [11–13].

There is inadequate information on the household socioeconomic status of people with SMD or depression in low-income countries due to methodological limitations of the studies to date. The few studies that have been conducted globally have linked SMD and depression to low socioeconomic status or poverty [2, 14, 15]. People with mental disorders have a lower income when compared to those without mental disorder [16], are more likely to live in rented and poor-quality housing [17], have lower educational status [18, 19], suffer from greater indebtedness [20, 21], have greater food insecurity [22, 23], be less likely to have any savings [24] and more likely to live in poverty [15]. The effect of poor mental health can extend to reduced consumption of food, and lower expenditure on clothes and durable goods at the household level [25]. The limitation of most existing studies is that they are facility-based (in settings where most people with mental health problems do not access care), have been conducted in middle-income rather than low-income country settings and do not examine the relative impact of depression vs. SMD in the same study.

Sustainable Development Goals one and 10 give strong emphasis to the importance of equitable development, equal rights to economic resources and the inclusion of marginalized and vulnerable groups within the population [26]. The inconsistent and limited evidence base regarding poverty and mental health in low-income country population samples limits advocacy efforts to ensure households with persons with mental health problems are given due attention. There is, therefore, a pressing need for high quality population-based studies using contextualized and comprehensive evaluations of socioeconomic status of households of persons with mental health problems living in low-income countries.

The study reported in this paper is part of the cross-country Emerald programme (Emerging mental health systems in low- and middle-income countries) which sought to provide rigorous, population-based evidence about the adequacy and fairness of mental healthcare financing [27]. This study was conducted at the baseline of the PRogramme for Improving Mental health care (PRIME), when no mental health care was available in the district; but mental health care was about to be integrated into primary care using a task-sharing model [28]. The objective was to compare the socio-economic status of households with persons with SMD or depression to households without these disorders in a rural Ethiopian setting.

## Methods

### Study design

A population-based, comparative, cross-sectional household survey was conducted in Sodo district, south Ethiopia, between January and August 2015 for the SMD cohort and March to November 2015 for the depression cohort.

### Setting

Sodo district is one of 15 districts in the Gurage Zone of Ethiopia. Administratively, the district is structured into 58 sub-districts (*kebeles*), with an estimated total population of 161,952, of whom around 90% reside in rural areas [29]. The economically active population is estimated to be 67.3%; 70.6% in the rural areas and 41.5% in urban areas [30]. The livelihoods of the population depend on agriculture, primarily farming and livestock rearing. During the data collection period, health services in the district were being delivered through eight health centers and 58 community health posts. The health post is staffed by community health extension workers and the health center by health officers, nurses, midwives, environmental officers and pharmacy technicians [31]. Sodo district was selected by PRIME and Emerald projects because it was mostly rural (in keeping with the rest of Ethiopia), included both highland and lowland topography, was accessible from Addis Ababa and neighbored a district where a psychiatric nurse-led out-patient unit was available [31].

### Sample size and participants

The sample size for the case cohorts were determined to address different objectives set within the PRIME and Emerald studies [23]. For the household cohorts, the sample was powered to detect a difference in household income. Comparison groups for the SMD study and the depression studywere recruited with a ratio of 1:1 to the corresponding case groups.

See Additional file 1: Figures S1 and S2 for the detailed recruitment procedure. For sample 1 (SMD study), community health extension workers, leaders, and lay data collectors who resided in the area were trained for half a day in typical presentations of SMD in this setting and then asked to identify people with possible SMD and refer them to the local primary care health centre. This approach to case ascertainment for SMD was used previously in the neighboring district and found to be sensitive [32]. The sample for the SMD study comprised community-ascertained people with possible SMD who attended the local health centre for treatment and were confirmed to have SMD (schizophrenia or other primary psychotic disorder, bipolar disorder or major depressive disorder with psychotic features) by psychiatric nurses using a semi-structured clinical interview (Operational Criteria for Research, OPCRIT) [33]*.* A comparison group of households was selected randomly from a sampling frame of a census of all households in the district which was carried out by PRIME [34]. The comparison household was matched to the household of the person with SMD on the basis of respondent characteristics (household head vs. other position in household), age (+/− 5 years), gender, *gott* (lowest level of residential area) and household size.

Sample two (depression study) comprised households of people attending the health centre who were identified by primary care staff as either having a probable diagnosis of depressive disorder or who screened positive on the Patient Health Questionnaire, nine item version (PHQ-9) and were thought to require treatment. The PHQ-9 was developed originally to identify probable depression in primary care samples in the US [35]. The PHQ-9 has been validated in Ethiopia in an urban teaching hospital out-patient clinic [36] and in primary care attendees in health centres in a district neighboring the location of the current study [37]. The comparison sample for the depression study comprised households of people who attended the health centre on the same day as the person with depression but who did not have a primary care worker diagnosis of depression and who had a PHQ score < 5, matched by gender, age (±5 years) and *gott*. The justification for selecting the comparison group from the facility rather than the community was so that the comparison sample was drawn from the base population that gave rise to the cases in order to minimize selection bias.

People attending the health centre with SMD, depression or no depression (facility comparison) were asked to give consent to visit their home. In the event that the person lacked capacity to consent, the caregiver was asked to give consent for the home visit. For the household interviews, the primary respondent was the household head, aged 18 years and above, who was willing to participate and provide consent. When the household head was absent for interview after three visits, a spouse or adult member of the household who was knowledgeable about the household economic status was interviewed.

### Measurements

#### Data collection and definitions

Outcome data were collected using an adapted and abbreviated version of the World Health Organization ERC-approved SAGE (Study of global AGEing and adult health) survey instrument. SAGE was used previously in a study on health and ageing in six LMICs [38].

Data on income and consumption expenditure were systematically collected for different time periods (weekly, monthly and annually) as applicable, and converted into standardized comparable time periods. Modifications were made to ensure contextual validity. For example, locally and culturally valued assets were identified for both urban and rural settings with the goal of distinguishing poor, middle and wealthier households.

Data collection from the case and the comparison households was conducted as close in time as possible, and not longer than 4 weeks apart.

#### Outcome variables (dependent variables)

Household socio-economic status was the primary outcome (income, consumption and asset possession). Socio-economic status refers to the social and economic factors that influence what position individuals or groups hold within the structure of a society [39]. Therefore, no single measure of socio-economic status will be ideal for all studies and contexts [39, 40]*.* Hence, in this study we aimed to examine multi-dimensional aspects of socio-economic status, based on household income, consumption and asset-based wealth.

Household income and consumption were estimated in terms of Ethiopian Birr and converted to US dollars (US$). The average 2015 exchange rate of 20.69 Ethiopian Birr (ETB) to US$1 was used [41]. In order to account for differences in household size and composition, total household income and consumption were made equivalent (‘equivalised’) using the OECD-modified scale [42, 43]. Using the household headcount, the scale assigns a value of 1 to the household head, of 0.5 to each additional adult member and of 0.3 to each child [42, 43]. By dividing household income and consumption by the OECD scale, these equivalised values can be expressed and compared based on income and consumption per adult equivalent.

The multi-dimensional measures of socioeconomic status used in this study were estimated as follows:

I. Household income from a range of sources (i.e., income from wages, rental property, trade, farming, savings and grants, transfers from families, community groups, and government and from other sources) were summed and adjusted for household size and composition using the OECD-modified scale [42, 43]. The resulting household income was categorized into five quintiles.
II. Household consumption (i.e., consumption of food produced by the household or purchased in the market place or given in kind to the household, consumption of non-food items for daily use, consumption of consumer durables, consumption of health care goods, consumption related to transfers out to household or community) were summed for each household and adjusted for household size and ageusing the OECD-modified scale. The resulting household consumption was then classified into five consumption quintiles.
III. An asset-based index was constructed on the basis of multiple correspondence analyses (MCA) using 35 items that included housing characteristics and sanitation facilities, access to basic services, ownership of a range of durable assets, household amenities and livestock. MCA is used to analyze a set of observations described by a set of nominal and quantitative variables and imposes fewer constraints on the data compared to principal component analysis [44].

### Primary exposure variable

The primary exposure was whether a household included a person with a mental health problem (SMD or depression) or not (comparison households).

### Covariates

Demographic variables included: household size, age, sex, marital status of the household head, and *gott*. Education was measured in terms of literacy, completed years of formal education and qualifications attained. Household size was measured by the total number of people who lived in the household (defined as those people who usually stay in the household, share meals (eat out of the same cooking pot), spends more than 4 months a year living there, or who usually stay there but are away currently for a short time.

Household composition reflects the number of children < 15 years and the number of adults in the household. This information used to make each member equivalent by weighting each according to their age, using the so-called modified OECD equivalence scale [42, 43].

The head of a household is a person who economically supports or manages the household or, for reasons of age or respect, is considered as a head by members of the household or declares themselves to be head of a household. The head of a household could be male or female.

### SMD and depression severity measures

#### WHODAS-ii

Disability was assessed using the 36-item fully structured interviewer administered version of the World Health Organization Disability Assessment Schedule second version (WHODAS–II) [45]. WHODAS–II assesses the level of disability and the number of days lost from work in the past 30 days due to health conditions. The WHODAS-II consists of six domains: mobility, self-care, life activities, understanding and communicating, interpersonal interactions, and participation in society. Total WHODAS-II scores range from zero to 100, with higher numbers indicating greater impairment of day-to-day functioning [45]. The Amharic version of this instrument was validated for people with SMD in Ethiopia previously [46–48]. For this study, people enrolled in the SMD or depression cohorts were classified into two groups based on the median of the WHODAS-II polytomous summary score, with the hypothesis that greater disability would be associated with greater socioeconomic disadvantage: Hence, for SMD cohort (A) higher disability (WHODAS-II score ≥ 52.7); (B) lower disability (WHODAS-II score < 52.7). For the depression cohort (A) Depression with higher disability (WHODAS- II score ≥ 33.3); (B) Depression with lower disability (WHODAS-II score < 33.3).

### Data collection and training

The household socioeconomic interview was administered by lay interviewers who had completed secondary school education and were experienced in data collection. The lay interviewers received 2 weeks of practical and theoretical training. The data collection was closely supervised and directly observed in the field by trained supervisors.

### Statistical analysis

The pre-coded responses were double entered by trained personnel using Epidata version 3.1 [49]. Analyses of variance (with the Scheffe’ test), Kruskal-Wallis and Pearson chi-squared test statistics were used, as appropriate, for testing unadjusted differences of mean, median and proportions to compare economic status between case and comparison households.

In multivariate regression, all estimates of household income and consumption expenditures (the dependent variables) were based on ordinal least squares regression of difference in outcome variables controlling for covariates. For asset-based wealth (the third dependent variable), estimates were based on an ordered logit regression model which adjusted for confounders. A negative coefficient represents lower income, consumption expenditure and asset-based wealth in relation to the comparison sample.

Both income and consumption data are highly skewed and so were log-transformed. The transformation ensures that errors are approximately homoscedastic. A Spearman rank correlation coefficient for the quintiles of consumption expenditure with asset-based wealth and with income was calculated. 0.36 and 0.35 for SMD, respectively. For the depression study, the corresponding values were0.25 and 0.43, respectively. The proportion of households classified in the same quintile by the three measures (consumption, income and asset quintiles) in the SMD study and depression study were 30.9 and 27.1%, respectively. Nevertheless, these measures are not equivalent and might represent different concepts of economic status in the study settings.

An exploratory examination of data for missing cases, outliers, and fulfillment of test assumptions was conducted as follows:

In the model of log income, the Breush-Pagan test for heteroscedasticity was carried out. In case of the presence of heteroscedasticity, Stock and Watson 2003, recommend use of heteroscedasticity-robust standard errors [50]. An important assumption for the multiple regression models is that independent variables are not perfectly multicollinear. Hence, the VIF (Variance Inflation Factor) was calculated.

Testing for omitted variable bias is important for our model since it is related to the assumption that the error term and the independent variables in the model are not correlated (E (e|X) = 0). Therefore, Ramsey RESET test using powers of the fitted values of the three economic outcomes was tested. Cameron and Trivedi’s [51] decomposition of information matrix (IM) for heteroscedasticity, skewness and kurtosis were also calculated. Data analysis was performed using STATA 13.1 (StataCorp LP, College Station, TX, USA) [52].

## Results

### Sample characteristics

The survey covered a total of 836 households: 290 households of persons with SMD (148 with higher disability and 142 with lower disability) and 289 comparison households without SMD, and 128 households of persons with depression (65 with higher disability, 63 with lowerdisability) and 129 comparison households without depression.

### General descriptive statistics

Table 1 presents descriptive results of households and clinical characteristics disaggregated by case, comparison and level of disability.

**Table 1:** Background characteristics of the Study Participants (Households) by Mental Health Conditions and Severity

Household Characteristics	Severe mental disorder (SMD) study			Depression study		
	Households of persons with SMD (*N* = 290)		Comparison households without persons with SMD (*N* = 289)	Households of person with depression (*N* = 128)		Comparison households for depression (*N* = 129)
	SMD with higher disability (*N* = 148)	SMD with lower disability (*N* = 142)		Depression with higher disability (*N* = 65)	Depression with lower disability (*N* = 63)	
Clinical Characteristics						
Functioning measured using						
WHODAS complex score Median (IQR)	69.4 (61.1, 80.5)***	30.5 (19.4, 41.6)***	5.5 (0.0, 19.4)	47.2 (38.9, 61.1)	16.6 (11.1, 25.0)	–
WHODAS simple score, Median (IQR)	32.0 (28.0, 36.5)***	13.5 (8.0, 18.0)***	2.0 (0.0,7.0)	20.0 (9.0, 26.0)	7.0 (6.0, 10.0)	–
Symptom scores						
BPRS-E,median (IQR)	48.0 (37.0, 59.0)	45.0 (34.5, 57.0)	–	–	–	–
PHQ-9, median (IQR)	–	–	–	12.0 (9.0, 15.0)	9.0 (6.0, 11.0)	–
Socio-demographic variables						
Household size, mean (SD)	5.1 (2.1)	5.2 (2.3)	5.2 (2.0)	5.2 (2.1)	4.9 (1.9)	5.0 (2.0)
Household composition,†† Mean (SD)	2.6 (0.8)	2.7 (0.9)	2.7 (0.8)	2.7 (0.9)	2.6 (0.8)	2.6 (0.8)
Rural residence, n (%)	118 (79.7)	117 (82.3)	236 (81.6)	46 (70.8)	57 (90.5)*	103 (79.8)
Gender of household head, n (%) Male	104 (70.7)	106 (74.6)	223 (77.9)	52 (80.0)	49 (80.3)	96 (75.0)
Age (years) of household head, mean (SD)	50.1(14.8)	48.8 (13.6)	49.9 (13.9)	48.7 (11.8)	44.1 (12.9)	44.2 (13.8)
Marital status of household head, n (%) married	101 (68.7)*	104 (73.2)	223 (77.7)	52 (80.0)	50 (83.3)	98 (76.6)
Educational level of household -head, n (%) no formal education	98 (66.2)*	87(61.2)	179 (61.9)	44 (67.6)	38 (60.3)	67 (52.3)

* *P* < 0.05, *** *P* < 0.001; **††** adult equivalent

*WHODAS (World Health Organization Disability Assessment Scale); IQR (Inter-Quartile Range); SD (Standard Deviation); BPRS-E (Brief Psychiatric Rating Scale, Expanded version); PHQ-9 (Patient Health Questionnaire item-9)*

### Clinical characteristics of the sampled population

The median (and interquartile range; IQR) of WHODAS-II complex score for households of persons with SMD with higherdisability (69.4; 61.1, 80.5) and lower disability (30.5; 19.4, 41.6) were considerably higher than households without a person with SMD (median WHODAS-II 5.5; IQR 0.0, 19.4); (χ^2^(2) test-for-trend =386.726; *p* < 0.001). In the depression study, the median (IQR) WHODAS-II score for depression with higher disability was 47.2 (38.9, 61.1) and for lower disability was 16.6 (11.1, 25.0); Pearson (χ^2^(1) = 102.7938; *p* < 0.001.

The median (IQR) depression symptom scores (PHQ-9 total) in the households of persons with higher and lower- disability depression were 12.0 (9.0, 15.0) and 9.0 (6.0, 11.0), respectively.

### Household characteristics

The probability of having an average household size was similar for all groups, ranging between 4.9 and 5.2. Respondents from households of persons with SMD with higher disability were less likely to be married or to have attended formal education than comparison households.

### Housing, water and sanitation facilities

A lower percentage of households of a person with SMD with lower disability owned their home (82.3%; *p* = 0.039) compared to households without a person with SMD (88.6%). See Additional file 2: Table S1. Households with a member with depression with higher disability were more likely to obtain drinking water from an unprotected source (38.5%; *p* = 0.045) compared with households without depression (28.7%). In 23% (*p* < 0.001) of households with persons with SMD with higher disability there was no toilet facility compared to 11.0% for households without a person with SMD.

### Household income -based measures

The results shown in Table 2 present the reliability and source of household income. Higher proportions [6.8% (95% CI, 3.7,12.3)] of households with SMD with higher disability reported not to have any income (*p* = 0.011). Relying on regular income was significantly lower for households with SMD with higher disability (*p* = 0.008) or depression with higher disability (*p* = 0.046) than households without a person with a mental health problem.

**Table 2:** Household’s income and consumption-based Welfare Measures by Mental Health Conditions and Severity

Household’s economic variables	Severe mental disorder (SMD) study			Depression study		Comparison households for depression (*N* = 129)
	Households of persons with SMD (*N* = 290)		Comparison households without persons with SMD (*N* = 289)	Households of persons with depression (*N* = 128)		
	SMD with higher disability (*N* = 148)	SMD with lower disability (*N* = 142)		Depression with higher- disability (*N* = 65)	Depression with lower disability (*N* = 63)	
Reliability of income, %(95% CI)						
Regular income	54.4**(46.2, 62.4)	57.7(49.4, 65.6)	66.2(60.5,71.4)	44.6*(32.9,56.9)	71.4(58.9,81.3)	63.2(54.5,71.2)
Seasonal income	38.6 (31.0, 46.8)	38.7(31.0, 47.0)	32.8(27.5,38.4)	52.3(40.1,64.2)	28.6(18.6,41.0)	34.3(26.6,43.0)
No income	6.8*(3.7, 12.3)	3.5(1.4, 8.2)	1.0 (0.3,3.2)	3.0(0.7,11.6)	0.0(0.0)	2.3(0.7,7.0)
Sources of income,%(95% CI)						
Wages	27.8(21.2, 35.7)	19.1.(13.4,26.5)	21.8(17.4, 27.0)	27.6(18.0,39.8)	12.7*(6.4,23.5)	27.9(20.7,36.3)
Trading	73.4**(65.7,80.0)	79.5*(72.1, 85.4)	87.5(83.1,90.8)	78.4(66.6,86.8)	90.4(80.2,95.7)	85.2(80.2,95.7)
Rental properties	12.8* (8.3, 19.2)	13.3*(8.6, 20.0)	6.2(3.9,9.6)	9.2(4.1,19.2)	3.1(0.7,12.0)	6.2(3.1,11.9)
Annual income HC, median (IQR) ‡	2584.4** (1316.0, 4651.5)	2888.8 (1666.6, 5313.2)	3654.7 (2000.0,5776.5)	3130.4* (2476.1,4761.9)	4193.5 (2400.0,6086.9)	4216.2 (2333.3,8333.3)
Annual consumption HC, median (IQR) ‡	8235.2 (4957.6, 12507.7)	8352.2 (5220.7, 12492.4)	8527.4 (5855.3, 13160.0)	7650.0** (5251.0,10737.5)	10054.1(6628.9, 16460.6)	10254.2 (7026.6, 16104.0)

‡ = Birr; US$1 = Birr 20.69 (2015);* *P* < 0.05, ** *P* < 0.01, *** *P* < 0.001; IQR (inter-quartile range); HC (headcount)

The median equivalised annual income earned by households of persons with SMD with lower disability was about 1070 Birr (51.7 USD) lower (*p* < 0.001) compared with households without a person with SMD. In the depression study, equivalized annual income was significantly lower (*p* = 0.047) by 1086 Birr (52.5 USD) for households with persons with depression with higher disability compared to control households without a person with depression.

After adjusting for household level socio-demographic characteristics, there was a clear association between levels of disability and household income (Table 3). The mean difference in income level of households of persons with SMD with higher disability and lower disability were *β* = − 0.325 (*p* = 0.005) and *β* = − 0.180 (*p* = 0.010), respectively, compared to the mean income level of households without persons with SMD. The mean annualincome of households of persons with depression with higher disability was lower (*β* = − 0.133; *p* = 0.042), compared to households without a person with depression.

**Table 3:** Regression coefficient estimate of log of income by mental health conditions, severity and covariates

(a). Severe mental disorder (SMD) study			
Characteristics	N(%), or mean (SD)	Household log of income	
		*unadjusted model*	*adjusted model*
Mental health conditions (%)		*β* (95% CI)	*β* (95% CI)
Households of SMD with higher disability	148 (25.7)	−0.295* (− 0.519, 0.071)	− 0.325** (− 0.547, 0.102)
Households of SMD with lower disability	141 (24.4)	− 0.155* (− 0.377, 0.066)	−0.180* (− 0.406, 0.045)
Households without SMD	288 (49.9)	1.00	1.00
Household characteristics^*†*^			
Male household head	431(75.4)	0.320** (0.093, 0.546)	0.271*(0.037, 0.506)
Age (years) of household head, mean(SD)	49.1(14.1)	−0.009**(− 0.015, 0.003)	− 0.009 **(− 0.015, 0.002)
Urban residence	108 (18.7)	0.192 (− 0.075, 0.459)	0.212 (− 0.073, 0.497)
Household head with no formal education	363 (63.1)	− 0.182 (− 0.453, 0.089)	−0.183 (− 0.446, 0.078)
Total adults*††*, mean (SD)	3.4 (1.4)	0.052 (− 0.025, 0.131)	0.076*(0.001, 0.155)
(b). Depression study			
Characteristics	N(%), mean (SD)	Household log of income	
		*unadjusted model*	*adjusted model*
Mental health conditions, n (%)		*β* (95% CI)	*β* (95% CI)
Depression with higher disability	65 (25.3)	−0.384* (−0.675, 0.092)	−0.133* (− 0.604, 0.020)
Depression with lower disability	63 (24.5)	−0.126 (− 0.414, 0.161)	−0.053 (− 0.412, 0.179)
Comparison households for depression	129 (50.2)	1.00	1.00
Household characteristics^*†*^			
Male household head	197 (77.6)	0.147(−0.134, 0.430)	0.013 (− 0.258, 0.322)
Age (years) of household head, mean (SD)	45.3(13.2)	−0.013**(− 0.022, 0.003)	−0.047(− 0.013, 0.006)
Urban residence	51 (19.8)	0.074 (− 0.235, 0.384)	0.010 (− 0.309, 0.360)
Household head with no formal education	149 (58.2)	−0.634**(−1.013, 0.255)	−0. 253* (− 0.924, 0.84)
Total adults*††*, mean (SD)	3.1(1.4)	−0.058 (− 0.152, 0.035)	− 0.004 (− 0.098, 0.092)

* P < 0.05, ** P < 0.01; SD (standard deviation); ***††*** *(headcount);* ^*†*^ *CI, confidence interval; Reference group for household characteristics (female household head, rural residence, and more than primary education). All estimates are based on OLS regression of change in outcome variables, controlling for covariates. Coefficient of a specific disorder (SMDs or depression) is from a separate OLS regression*

**Table 4:** Regression coefficient of log of consumption by mental health conditions, severity and covariates

(a) Severe mental disorder (SMD) study			
Characteristics	N (%), mean (SD)	Household log of consumption	
		*unadjusted model*	*adjusted model*
Mental health conditions, n (%)		*β* (95% CI)	*β* (95% CI)
Households of SMD with higher disability	148 (25.6)	− 0.058 (− 0.200, 0.085)	−0.106 (− 0.509, 0.273)
Households of SMD with lower disability	141 (24.4)	−0.033 (− 0.184, 0.116)	−0.010 (− 0.152, 0.172)
Households without SMD	289 (50.0)	1.00	1.00
Household characteristics^*†*^			
Household Composition*††*, mean (SD)	2.7 (0.9)	0.091*(0.021, 0.162)	0.030 (−0.051, 0.112)
Male household head	433(75.4)	0.108 (−0.027, 0.244)	0.063 (− 0.061, 0.188)
Urban residence	108(18.7)	0.322*** (0.168, 0.476)	0.083 (−0.300, 0.184)
Household head with no formal education	363(62.7)	−0.155 (− 0.340, 0.029)	−0.198 (− 0.387, 0.010)
Log of income		0.248 ***(0.186, 0.309)	0.227 ***(0.145, 0.309)
Total debt***††***	341.2(974.5) ‡	0.0001*** (0.000, 0.00014)	0.0007* (0.00001, 0.00010)
Lowest Wealth	109(18.9)	−0.771***(− 0.946, − 0.595)	−0.684 ***(− 0.923, − 0.445)
Log of annual health expenditure		0.011 (− 0.060, 0.083)	- 0.013 (− 0.073, 0.047)
(b) Depression study			
Characteristics	N(%), or mean (SD)	Household log of consumption	
		*unadjusted model*	*adjusted model*
Mental health conditions (%)		*β* (95% CI)	*β* (95% CI)
Depression with higher disability	65 (25.3)	−0.288** (−0.491, − 0.085)	−0.122* (− 0.349, − 0.104)
Depression with lower disability	63 (24.5)	−0.023 (− 0.257, 0.209)	- 0.016 (− 0.157, 0.245)
Comparison households for depression	129 (50.2)	1.00^†^	1.00^†^
Household characteristics^*†*^			
Household Composition*††*,mean (SD)	2.6 (0.8)	−0.225***(− 0.339, 0.110)	−0.150 ***(− 0.292, − 0.081)
Male household head	197 (77.6)	0.082 (− 0.138, 0.303)	0.018 (− 0.236, 0.273)
Urban residence	51(19.8)	0.322*** (0.168, 0.476)	0.064 (− 0.317, 0.189)
Household head with no formal education	149 (58.2)	− 0.565***(− 0.827, − 0.302)	−0.256 (− 0.565, 0.052)
Log of income		0.323*** (0.217, 0.429)	0.201 **(0.088, 0.314)
Total debt*††*	536.2 (1396.5) ‡	0.00008*(0.00001, 0.00014)	0.00007* (0.00002, 0.00012)
Lowest Wealth		−0.577***(− 0.862, − 0.292)	−0.311**(− 0.684, 0.061)
Log of annual health expenditure		0.251*** (0.159,0.342)	0.209***(0.123, 0.294)

* P < 0.05, ** P < 0.01, *** P < 0.001; ‡ = Birr; US$1 = Birr 20.69 (2015); *CI, confidence interval*; *All estimates are based on OLS regression of change in outcome variables, controlling for covariate. Coefficient of a specific disorder (SMDs or depression) is from a separate OLS regression.* ***††*** *HC (headcount);* ^*†*^ *Reference group for household characteristics (female household head, rural residence, and more than primary education)*

In the model of log income, the Breush-Pagan test for heteroscedasticity indicated possible heteroscedasticity in both the SMD and depression models (*p* = 0.1 and 0.3). The mean Variance Inflation Factor was 1.57 for the SMD regression model and 1.50 for the depression model, indicating no effect of multicollinearity.

The Ramsey RESET test indicated that the model has no omitted variables (*p* = 0.68 for SMD model and 0.27 for depression). Cameron and Trivedi’s [51] decomposition of information matrix for heteroscedasticity, skewness and kurtosis was 0.5, 0.5 and 0.01 for the SMD model and 0.9, 0.1 and 0.04 for the depression model, respectively.

### Household consumption-based measures

Table 2 shows that there were differences in median consumption expenditure in case vs. comparison households and with respect to levels of disability. Households of persons with SMD with higher disability and households of persons with depression with higher disability had lower consumption expenditure by Birr 292 (14.1 USD) and Birr 2604 (125.8 USD), respectively, compared to households without a person with a mental health problem. Table 4 shows the estimated ordinary least square (OLS) coefficients for the effect of SMD, depression and covariates, on household’s consumption expenditures. Households of a person with SMD with higher disability had 10.6 percentage points lower in consumption expenditures compared to households without SMD (β = -0.106, *p* > 0.05). In contrast, households of a person with depression with higher disability had 12.2% lower consumption expenditures compared to households without a person with depression (β = -0.122, *p* < 0.05). In the model of log-consumption heteroscedasticity, skewness, Kurtosis, VIF and Ramsey RESET test were (0.4 vs.0.5), (0.5 vs.0.6), (0.2 vs.0.1), (1.5 vs.1.8) and (0.3 vs.0.4) for SMD and depression models respectively.

### Asset ownership and asset-based wealth

A significantly lower percentage of households of persons with SMD with higher disability owned a mobile phone (41.2% vs. 57%; *p* = 0.006), radio (35.8% vs. 53.2%; *p* = 0.001), television (4.7% vs. 11.7%; *p* = 0.043), livestock (76.5% vs. 88.5%; *p* = 0.002), Table (23.4% vs. 36.3%; *p* = 0.008) or cassette recorder (13.4% vs. 23.1%; *p* = 0.018). See Additional file 2: Table S2. In the depression study, households of persons with depression with higher disability had significantly lower assets compared to households without a person with depression in terms of ownership of a television (*p* = 0.043), livestock (*p* = 0.026) and land (*p* = 0.035).

There was a difference between households in relation to asset-based wealth quintiles. Greater numbers of households of persons with SMD with higher disability were found in the lowest quintile (24.8% vs. 14.5%), compared to comparison households. In sharp contrast, a smaller percentage (17%; *p* = 0.007 and 15%; *p* = 0.003) of households of persons with SMD with higher disability or lower disability, respectively, were in the highest quintiles, compared to 26.3% in households without a person with a mental health problem. In the depression study we found similar differences across groups. The percentage of households of persons with depression with lower disability in the lowest quintile was 9.0% (*p* = 0.019) higher compared to comparison households without depression.

The results of the regression of asset-based wealth for SMD and depression cohorts are presented in Table 5. Households of persons with SMD with higher disability or lower disability owned significantly fewer assets, with mean differences of β = − 0.630 (*p* = 0.001) and β = − 0.54.2 (*p* = 0.005) compared to households without a person with SMD.

**Table 5:** Regression coefficient of asset based wealth by mental health conditions, severity and covariates

(a) Severe mental disorder (SMD) study			
Characteristics	N (%), or mean (SD)	Household asset based wealth	
		*unadjusted model*	*adjusted model*
Mental health conditions (%)		*β* (95% CI)	*β* (95% CI)
Households of SMD with higher disability	148 (25.6)	−0.536** (− 0.891, − 0.181)	−0.630 **(−1.000, − 0.252)
Households of SMD with lower disability	142(24.5)	−0.499** (− 0.853, − 0.145)	− 0.542** (− 0.916, − 0.167)
Households without SMD	289 (49.9)	1.00	1.00
Household characteristics^†^			
Male household head	433 (75.3)	−0.204 (− 0.536, 0.128)	−0.148 (− 0.506, 0.209)
Urban residence	108 (18.7)	2.936*** (2.465, 3.407)	2.990*** (2.501, 3.480)
Household head with no formal education	364 (62.9)	−0.047 (− 0.443, 0.538)	−0.059 (− 0.464, 0.584)
Log of income		0.449*** (0.306, 0.592)	0.451*** (0.300, 0.602)
Total debt*††*	341.2(974.5)‡	0.00005(−0.00009, 0.00020)	−0.0004 (− 0.002, 0.0011)
(b) Depression study			
Characteristics	N %, or mean (SD)	Household asset based wealth	
		*unadjusted model*	*adjusted model*
Mental health conditions		*β* (95% CI)	*β* (95% CI)
Depression with higher disability	65 (25.3)	−0.643* (−1.173, − 0.112)	− 0.227 (− 0.793, 0.338)
Depression with lower disability	63 (24.5)	−0.003 (− 0.522, 0.529)	− 0.020(− 0.606, 0.565)
Comparison households for depression	129 (50.2)	1.00	1.00
Household characteristics^†^			
Male household head	197 (77.6)	0.284(−0.234, 0.802)	0.205 (− 0.355, 0.767)
Urban residence	51 (19.8)	3.171*** (2.463, 3.879)	3.159*** (2.468, 3.850)
Household head with no formal education	149 (58.2)	−1.523*** (−2.172, −0.874)	− 1.170 **(− 1.871, − 0.469)
Log of income		0.548***(0.303,0.793)	0.215*** (0.111, 0.320)
Total debt*††*	536.2(1396.5) ‡	−0.00008(− 0.00021, 0.00005)	− 0.00009 (− 0.00021, 0.00003)

* *P* < 0.05, ** *P* < 0.01, ***P < 0.001; *‡ = Birr; US$1 = Birr 20.69 (2015);* ***††*** *HC (headcount);*^*†*^ *CI, confidence interval; Reference group; SD (standard deviation);* ^*†*^ *Reference group for household characteristics (female household head, rural residence, and more than primary education). All estimates are based on ordered logit regression of change in outcome variables. Coefficient of a specific disorder (SMD or depression) is from a separate ordered logit regression models*

There was no evidence of an interaction between SMD or depression and educational status, gender of the household head or residential area in relation to household income and consumption.

## Discussion

Consistent with theoretical expectations, the findings from this study demonstrate that households of persons with SMD or depression are economically disadvantaged in terms of income, consumption and possession of assets. Furthermore, in terms of living conditions and access to basic facilities, households with SMD or depression were significantly less likely to have a regular source of income, access to a toilet facility or access to water from a protected source. Households with persons with SMD or depression are, therefore, unable to meet essential requirements for their basic living and livelihoods.

After controlling for economic and demographic related variables, SMD and depression were associated with significantly lower household income. Moreover, there was a clear association between level of disability and household income, with a dose response seen between level of disability and level of socio-economic disadvantage compared to the households without a person with a mental health problem. Although this relationship is complex, higher disability is associated with impaired day-to-day functioning and reduced productivity [47, 53] which would be expected to have a downward effect on income.

The other possible reason for the lower income by households of persons with SMD or depression with higher or lower disability is the caregiver opportunity costs and perhaps an overall effect of stigma/social exclusion which may impede households’ income generation opportunitiesor their ability to draw on social networks for support. Previous research has shown that earnings and long-term work incapacity are both much more strongly associated with SMD compared with absence of SMD [54].

Our study result is consistent with a previous study from rural Ethiopia which found that severe mental disorder was associated with substantially lower income among households with a person with bipolar disorder when compared with control households with a person with a chronic physical disorder [7]. Furthermore, our findings are similar to other studies from LMICs. For example, the World Mental Health Survey that included bipolar disorder and depression [9] and SMD from South Africa [16] indicated an association between SMD or depression and reduced earnings. In a study from a high-income country, SMD was associated with the highest level of income-related inequality compared to the general population [4]. Jenkins et al. (2008) found that people with mental disorder had significantly less income, and more debt and financial hardship, than those without this disorder [21].

Equivalised household consumption in households with SMD with higher disability was lower than comparison households, but the difference was not significant. One explanation for this finding could be that consumption was already at the minimum level for survival [55] and so there was little scope for consumption to decrease significantly. It appears that depletion of assets occurred to maintain consumption in the face of reduced income.

In the depression study, similar to the work of Dahal et al. (2013) and Tampubolon and Hanandita (2014), we found lower consumption in households of persons with depression with higher disability [56, 57]. This could be through the pathway that depression induced reductions in income and labor supply that resulted in reduced consumption.

SMD and depression tend to be chronic or recurrent conditions that have the potential to impose high- and long-term economic burdens on households through asset depletion. As reflected in the asset index, we detected wealth difference in terms of position of households. A higher proportion of households of persons with SMD with higher disability were in the lowest asset-based wealth quintile. Similarly, a lower proportion of households of a person with depression with higher or lower disability were found in the highest asset quintile compared with households without depression.

Controlling for other variables, households of a person with SMD with higher or lower disability owned statistically significant fewer assets compared with their counterparts (*p* = 0.001) and (*p* = 0.005), respectively. However, households with a person with depression did not have significantly fewer assets than households without a person with depression. A possible explanation for this difference might be that SMD and depression affect household assets differently. Lower asset-based wealth in SMD may reflect chronicity of the financial impact of the illness, due to more difficulty with recovery and regaining capacity to produce assets. Although households with depression are disadvantaged economically, they may find it easier to regain their socio-economic status once the index person recovers so that the impact on asset depletion might be minimal.

Our results for SMD are in line with the findings by Yilma et al. (2014) on general illness that conclude illness is associated with asset depletion [58]. In a previous study from Ethiopia on maternal depression, higher disability scores were significantly associated with possessing fewer assets and self-rated lower wealth [53]. In general, the findings of lower income, lower consumption and possession of fewer assets by households of persons with SMD in our study are similar to the findings of Trani et al. (2015) in India that reported multidimensional poverty is linked to severe mental illness [15].

In line with expectations, the reported annual income was lower than consumption. This may be a true finding, reflecting that households use other means to maintain consumption levels in the face of financial stress or income shortfall, for example, borrowing money or using savings. The discrepancy may also reflect reporting bias for income, given the sensitive nature of the question. We argue that our study overcomes the limitations of previous studies. First, the study sample was community based and based on households that are representative of a sample population. Secondly, we used comprehensive measures of socioeconomic context. However, our study has several limitations: our analyses cannot show the causal effect of SMD or depression on economic status of households or vice versa. The cross-sectional study design does not allow us to elucidate the mechanisms behind the associations we have observed. Thus, prospective studies are needed. Furthermore, the existence of endogeneity cannot be ruled out although we have controlled for various demographic and economic variables. Respondents might not be comfortable about revealing information on income although confidentiality was assured. One other limitation of the study is that we do not know whether the comparison households for depression included persons with depression as we were not able to screen all household members.

## Conclusions

Households with a member who had a severe mental disorder or depression were socioeconomically disadvantaged compared to the general population. Therefore, we argue that mental disorders should be an important concern for development strategies and this vulnerable group should be prioritized with development assistance and access to evidence-based mental healthcare implemented by the government. Our study findings also indicate the need to further explore the economic impacts of different types of mental disorders at the household level across different African and LMIC settings.

## Additional files

Additional file 1: **Figure S1.** Patient recruitment flow for SMD study. **Figure S2.** Patient recruitment flow for depression study (DOC 38 kb)

Additional file 2: **Table S1.** Housing, Water and Sanitation Characteristics by Mental Health Conditions and Severity. **Table S2.**Household Asset Ownership and Asset Based Wealth Distribution by Mental Health Disorder and Severity (DOC 107 kb)

## Data Availability

The data are being used for a PhD student (YH) for his thesis and are not, therefore, available at the present time to the general public. The data may be requested from the corresponding author for verification of the analyses in this paper on a reasonable request.

## Abbreviations

BPRS-E: Brief psychiatric rating scale, Expanded version
Emerald: Emerging mental health systems in low- and middle-income countries
GDP: Gross domestic product
LMICs: Low- and middle-income countries
MCA: multiple correspondence analyses
OCRPIT: Operational criteria checklist for psychotic illness and affective illness
OECD: Organization of economic co-operation and development
OLS: ordinary least squares
PHQ-9: Patient health questionnaire – 9 item
PRIME: Programme for improving mental health care
SAGE: Study on global Ageing and adult health
SMD: Severe mental disorder
VIF: variance inflation factor
WHO: World Health Organization
WHODAS: WHO disability assessment schedule
