# Supplementary material for: Mental health problems and socioeconomic disadvantage: a controlled household study in rural Ethiopia
Source: Int J Equity Health. 2019 Jul 31;18:121. doi: 10.1186/s12939-019-1020-4 (PMC6670213; doi:10.1186/s12939-019-1020-4)
Supplement: Supplementary file 1 — Figure S1. Patient recruitment flow for SMD study. Figure S2. Patient recruitment flow for depression study (DOC 38 kb) [file 12939_2019_1020_MOESM1_ESM.doc]

Referred and attended PHC (N=467)

Comparison households without SMD identified from census(N=290)

PHC worker diagnosis of SMD (N=294)

Consents / Guardian assent for the study (N=304)

Visit household. Request consent to participate in economic study (N=290)

Diagnostic confirmation by psychiatric nurse (N=300)

Request consent from patient or caregiver to visit household within 2 weeks(N=300)

Administer household economic interview(N=289)

Community case-finding of SMD

PRIME cohort of

Individuals with SMD, receiving treatment

(n=300)

Visit household. Request consent to participate in economic study (N=290)

Administer household economic interview(N=290)

SMD cohort (N=290)

Cohort of comparison group without SMD(N=290)

Additional file 1: Figure S1: Patient recruitment flow for SMD study

No depression (N=135)

PHC worker diagnosis of depression/PHQ case positive(N=129)

Request consent to visit household (N=135)

Visit household. Request consent to participate in the study (N=129)

Visit household. Request consent to participate in the study (N=129)

Consenting (N=129)

Consenting (N=129)

Administer household economic interview (N=129)

Administer household economic interview (N=129)

PHC worker assessment

Request consent to visit household (N=129)

Comparison

Cohort(N=129)

Depression cohort (N=129)

Figure S2: Patient recruitment flow for depression study
