# Supplementary material for: Mental health problems and socioeconomic disadvantage: a controlled household study in rural Ethiopia
Source: Int J Equity Health. 2019 Jul 31;18:121. doi: 10.1186/s12939-019-1020-4 (PMC6670213; doi:10.1186/s12939-019-1020-4)
Supplement: Supplementary file 2 — Table S1. Housing, Water and Sanitation Characteristics by Mental Health Conditions and Severity. Table S2.Household Asset Ownership and Asset Based Wealth Distribution by Mental Health Disorder and Severity (DOC 107 kb) [file 12939_2019_1020_MOESM2_ESM.doc]

**Additional file 2: Table S**1. Housing, Water and Sanitation Characteristics by Mental Health Conditions and Severity

| **Household Characteristics** | **Severe mental disorder (SMD) study** | | | **Depression study** | | |
| --- | --- | --- | --- | --- | --- | --- |
|  | **Households of persons with SMD**  **( N=290)** | | **Comparison households without persons with SMD**  **( N = 289)** | **Households of persons with depression**  **( N=128)** | | **Comparison households**  **for depression**  **( N= 129)** |
|  | **SMD with higher disability**  ( N= 148) | **SMD with lower disability** (N= 142) |  | **Depression with higher disability**  **( N=65)** | **Depression with lower disability**  **( N=6 )** |  |
| **Housing** | **N (%)** |  | **N (%)** | **N (%)** | **N (%)** | **N (%)** |
| Housing ownership: owns house fully paid off | 128(85.9) | 117(82.3)* | 256 (88.6) | 51 (78.4) | 51 (80.9) | 103 (79.8) |
| Roof material: corrugated iron roofing | 82 (55.0) | 76 (53.9) | 158 (54.6) | 45 (69.2)* | 29 (46.0) | 79 (61.2) |
| Floor material: hard floor | 1 (0.67) | 5 (3.6) | 7 (2.4) | 1 (1.5) | 2 (3.1) | 10 (7.8) |
| Wall material: wood and mud wall | 147 (99.3) | 139 (98.5) | 284 (98.2) | 65 (100.0) | 61 (96.8) | 122 (95.3) |
| Proxy for household crowding:  Headcount per room, mean (SD) **††** | 2.0 (1.0) | 2.1(0.9) | 2.0 (0.9) | 1.8 (1.0) | 2.0 (1.0) | 1.8 (0.9) |
| Cooking in room used for sleeping and living | 50(33.5)* | 45 ( 32.1) | 70 (24.3) | 15 (23.0) | 19 (30.1) | 28 (22.0) |
| **Water and Sanitation** |  |  |  |  |  |  |
| Source of drinking water |  |  |  |  |  |  |
| Piped | 12(8.0) | 15(10.6) | 30 (10.3) | 8 (12.3) | 6 (9.5) | 12 (9.3) |
| Public stand point | 46 (30.8) | 48 (34.0) | 97 (33.5) | 25 (38.4) | 30 (47.6) | 76 (58.9) |
| Protected spring or well | 11 ( 7.3) | 15(10.6) | 21 (7.2) | 7 (10.8) | 3 (4.7) | 4 (3.1) |
| Unprotected source (river) | 80 (53.6) | 63 (44.6) | 141 (48.7) | 25 (38.5)* | 24 (38.1) | 37 (28.7) |
| More than 30 minutes to access water from protected source | 80 (53.6) | 63 (44.6) | 141 (48.7) | 25 (38.5) | 24 (38.1) | 37 (28.7) |
| Average time to access water in minutes, mean (SD) | 36.0 (34.8) | 31.9 (31.6) | 30.9 (40.0) | 25.4 (24.2) | 28.1 (24.0) | 28.2 (23.2) |
| **Toilet facility** |  |  |  |  |  |  |
| No facility | 34 (22.9)** * | 36 (25.5) ** | 32 (11.1) | 13 (20.0) | 12 (19.0) | 18 (14.0) |
| Pit Latrine (without slab) | 106 (71.7) | 101 (71.6) | 244 (84.4) | 49 (75.4) | 48 (76.1) | 103 (80.5) |
| Flush or VIP latrine | 8(5.4) | 4 (2.9) | 13 (4.5) | 3 (4.6) | 3 (4.7) | 7 (5.5) |

** P<0.05, *** P<0.001;* ***††*** *adult equivalent*

*Protected source= (piped, public stand point, protected spring or protected well)*

**Table S**2.Household Asset Ownership and Asset Based Wealth Distribution by Mental Health Disorder and Severity

| **Asset ownership** | **Severe mental disorder (SMD) study** | | | **Depression study** | | |
| --- | --- | --- | --- | --- | --- | --- |
|  | **Households of persons with SMD**  **( N=290)** | | **Comparison households without persons with SMD**  **( N=289)** | **Households of persons with depression ( N=128 )** | | **Comparison households for depression**  **( N= 129 )** |
|  | **SMD with higher disability**  **( N= 148)** | **SMD with lower disability (N= 142)** |  | **Depression with higher disability**  **( N=65)** | **Depression with lower disability**  **( N=63)** |  |
| **Possession of assets** | **N (%)** | **N (%)** | **N (%)** | **N (%)** | **N (%)** | **N (%)** |
| Electric/solar supply | 38 (25.6) | 37 (26.0) | 79 (27.4;) | 25 (38.5) | 18 (29.0) | 53 (41.1) |
| Cell phone | 61 (41.2)** | 71 (50.0) | 165 (57.0) | 39 (60.0) | 45 (71.4) | 89 (69.0) |
| Radio | 53 (35.8)** | 59 (41.5)* | 154 (53.2) | 38 (58.5) | 36 (57.1) | 70 (54.3) |
| Watch clock | 26 (17.4) | 29 (20.5) | 65 (22.4) | 15 (23.1) | 12 (19.0) | 40 (31.0) |
| Television | 7 (4.7)* | 9 (6.3) | 34 (11.7) | 2 (3.0)* | 2 (3.1) | 14 (10.9) |
| Bicycle | 4 (2.6) | 2 (1.4) | 6 (2.0) | 0 (0.0) | 0 (0.0) | 6 (4.6)* |
| Car/Truck | 0 (0.0) | 0 (0.0) | 1 (0.3) | 1 (1.5) | 0 (0.0) | 1 (0.8) |
| Motorbike | 0 (0.0) | 1 (0.7) | 2 (0.6) | 0 (0.0) | 0 (0.0) | 2 (1.5) |
| Landline phone | 4 (2.6) | 3 (2.1) | 12 (4.1) | 3 (4.6) | 2 (3.1) | 7 (5.4) |
| Land | 128 (85.9) | 129 (91.4) | 250 (86.5) | 46 (70.8)* | 58 (92.0) | 102 (79.1) |
| Animal drawn carts | 7 (4.7) | 8 (5.6) | 25 (8.6) | 8 (12.3) | 7 (11.1) | 12 (9.3) |
| Wooden bed | 38 (25.6) | 38 ( 26.9) | 98 (33.9) | 21 (32.3) | 18 (28.5) | 45 (34.9) |
| Table | 35 (23.4)** | 43 (30.5) | 105 (36.3) | 23 (35.3) | 16 (25.4) | 51 (39.5) |
| Chair | 134 (89.9)* | 131 (92.9) | 277 (95.8) | 61 (93.8) | 59 (93.6) | 124 (96.1) |
| Wooden shelf | 50 (33.5) | 46 (32.6) | 109 (37.7) | 29 (44.6) | 17 (26.9) | 41 (31.8) |
| Cupboard | 8 (5.3) | 7 (5.0) | 28 (9.6) | 6 (9.2) | 3 (4.7) | 12 (9.3) |
| Any livestock | 114 (76.5)** | 113 (80.1)* | 256 (88.5) | 46 (70.8)* | 55 (87.3) | 108 (84.3) |
| Cow/oxen | 93 (81.5)* | 100 (89.2) | 231 (88.5) | 43 (66.1) | 50 (79.3) | 94 (72.8) |
| Goat/sheep | 61 (53.1)* | 62 (54.8)* | 173 (66.5) | 30 (46.8) | 31 (49.2) | 73 (56.5) |
| Mule/donkey/horse | 57 (50.0)* | 66 (58.4) | 167 (63.9) | 32 (49.2) | 37 (58.7) | 70 (54.2) |
| Chicken | 78 (68.4) | 77 (68.1)* | 190 (72.8) | 32 (49.2) | 36 (57.1) | 78 (60.4) |
| Bucket/pot | 145 (97.3) | 136 (96.4) | 287 (99.3) | 64 (98.4) | 63 (100.0) | 127 (98.4) |
| Grass/animal hide mattress | 44 (29.5) | 40 (28.3) | 105 (36.3) | 24 (36.9) | 21 (33.3) | 44 (34.1) |
| Kerosene stove/coal pot | 62 (41.6) | 59 (41.8) | 135 (46.7) | 33 (50.7) | 26 (41.2) | 73 (56.5) |
| Cassette recorder | 20 (13.4)* | 21 (14.8)* | 67 (23.1) | 13 (20.0) | 19 (30.1) | 37 (28.6) |
| Sponge/cotton mattress | 45 (30.2) | 42(29.7) | 97 (33.5) | 24 (36.9) | 17 (26.9) | 48 (37.2) |
| Paraffin/gas stove | 4 (2.6) | 4 (2.8) | 11 (3.8) | 1 (1.5) | 0 (0.0) | 12 (9.3)* |
| Sofa chair | 2 (1.3) | 6 (4.2) | 13 (4.5) | 5 (7.7) | 0 (0.0) | 7 (5.4) |
| Satellite dish | 5 (3.3) | 6 (4.2) | 21 (7.2) | 1 (1.5) | 2 (3.2) | 10 (7.7) |
| CD player | 5(3.3) | 7 (4.9) | 18 (6.2) | 1 (1.5) | 2 (3.1) | 8 (6.2) |
| **Asset quintile, n (%)** |  |  |  |  |  |  |
| Lowest | 37 (24.8)** | 30 (21.2)** | 42 (14.5) | 11 (16.9) | 17 (26.9)* | 23 (17.8) |
| Low | 28 (18.7) | 34(24.1) | 51 (17.6) | 12 (18.4) | 14 (22.2) | 25 (19.3) |
| Middle | 28 (18.7) | 29 (20.5) | 62 (21.4) | 12 (18.4) | 17 (26.9) | 23 (17.8) |
| High | 30(20.1) | 26(18.4) | 58 (20.0) | 17 (26.1) | 6 (9.5) | 28 (21.7) |
| Highest | 26(17.4)* | 22 (15.6)** | 76 (26.3) | 13 (20.0) | 9 (14.3)* | 30 (23.2) |

** P<0.05, ** P<0.01, **, P<0.001,*
